# Supplementary figures and images for: Discovering disease genetic variation impacting gene expression in 103 brain tissues with the Brain Ontology Expression (BRONTE) graph neural network model
Source: bioRxiv. 2025 May 14:2025.05.08.652857. Preprint. [Version 1] doi: 10.1101/2025.05.08.652857 (PMC12132177; doi:10.1101/2025.05.08.652857)

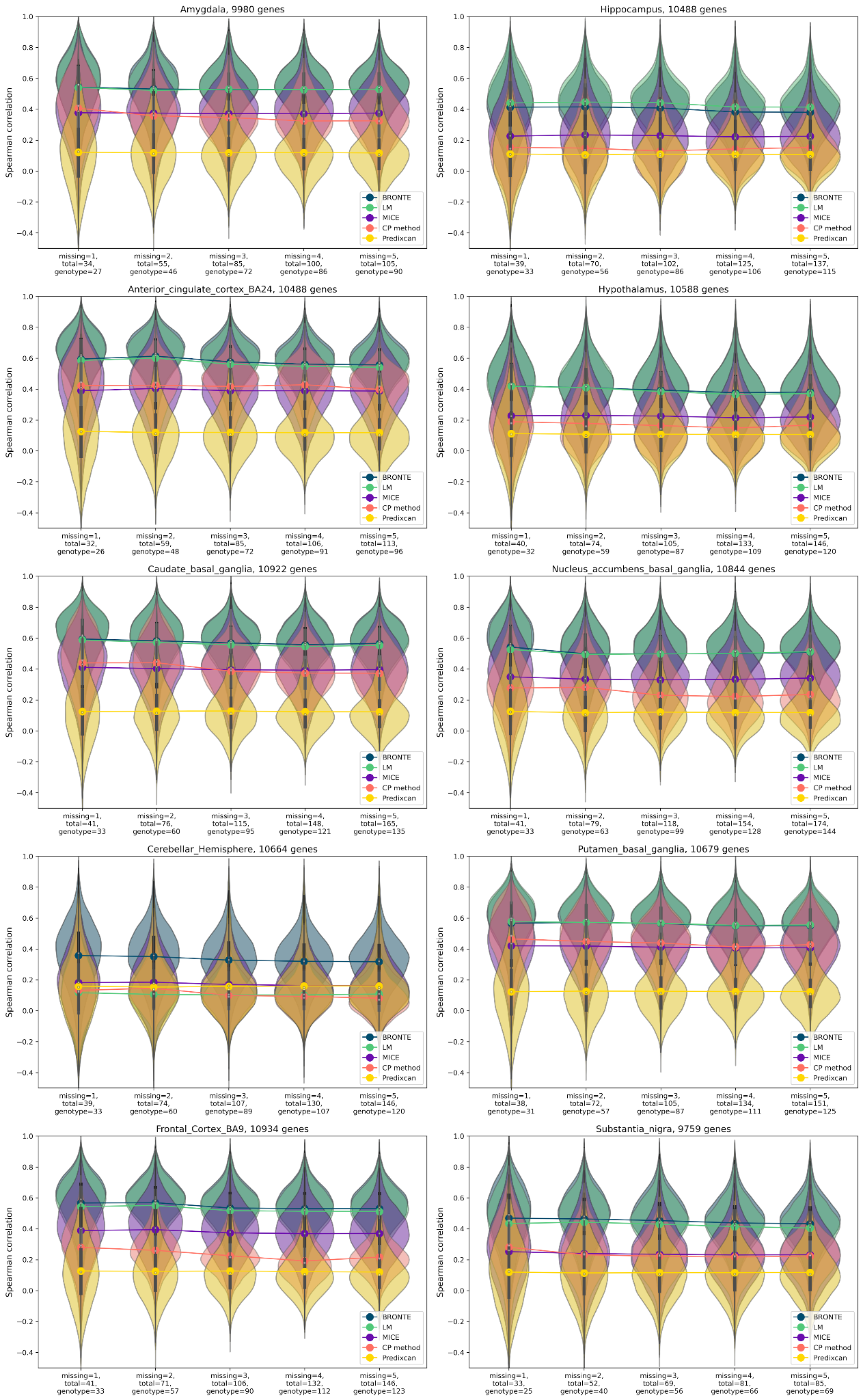

Supplement: Supplement 1 [file media-1.zip › Supplementary Figure 1.docx]

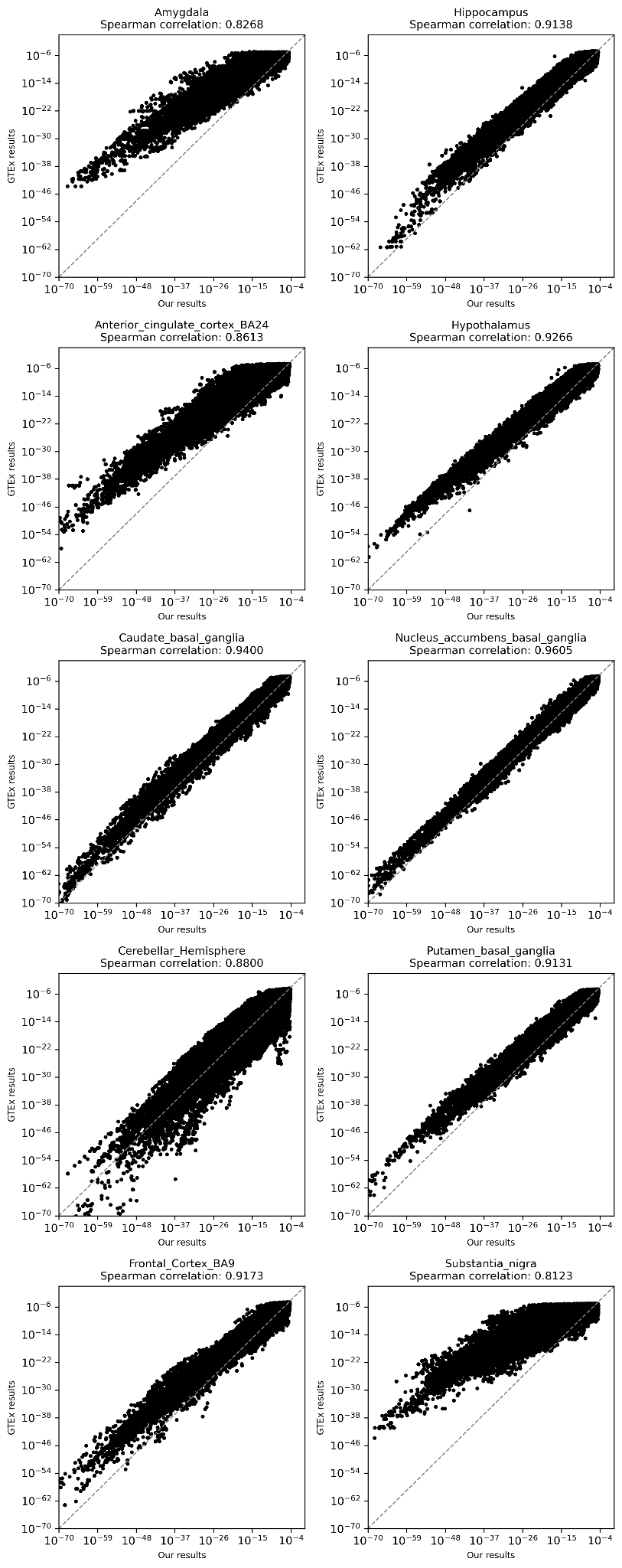



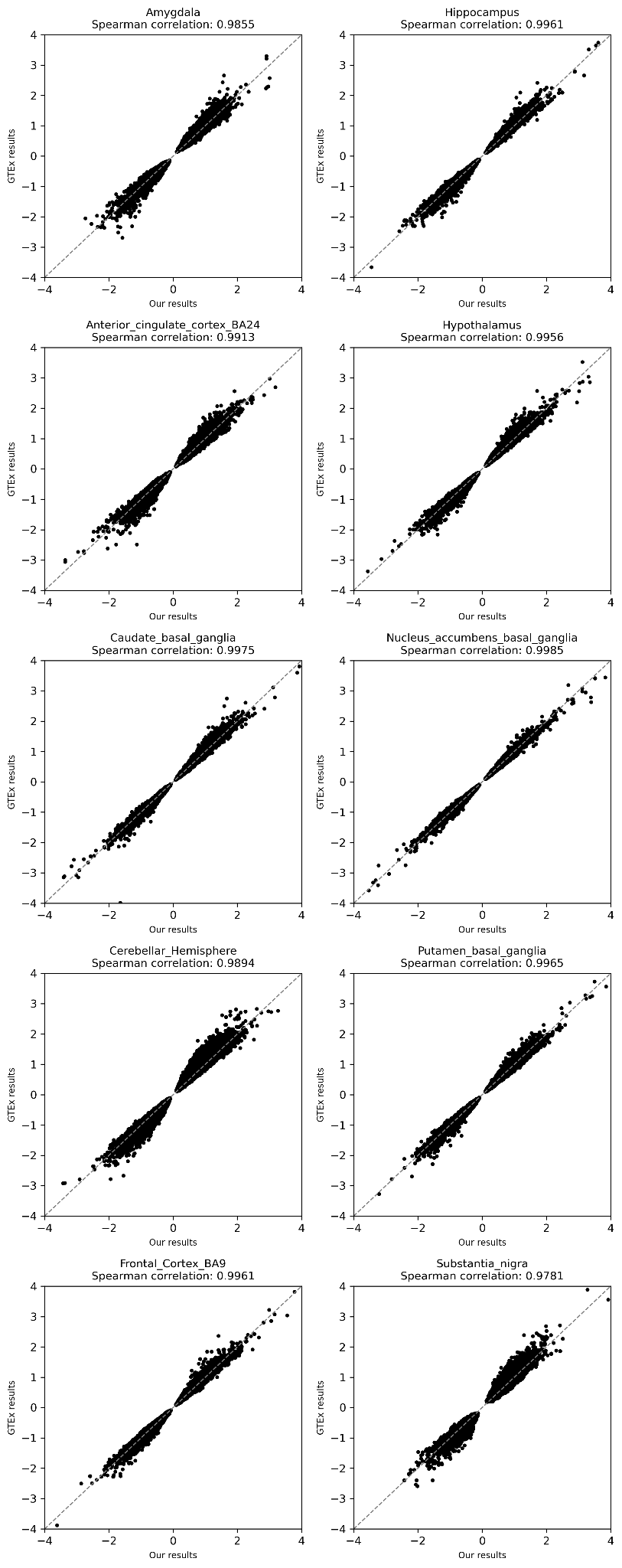

Supplement: Supplement 1 [file media-1.zip › Supplementary Figure 2&3.docx]

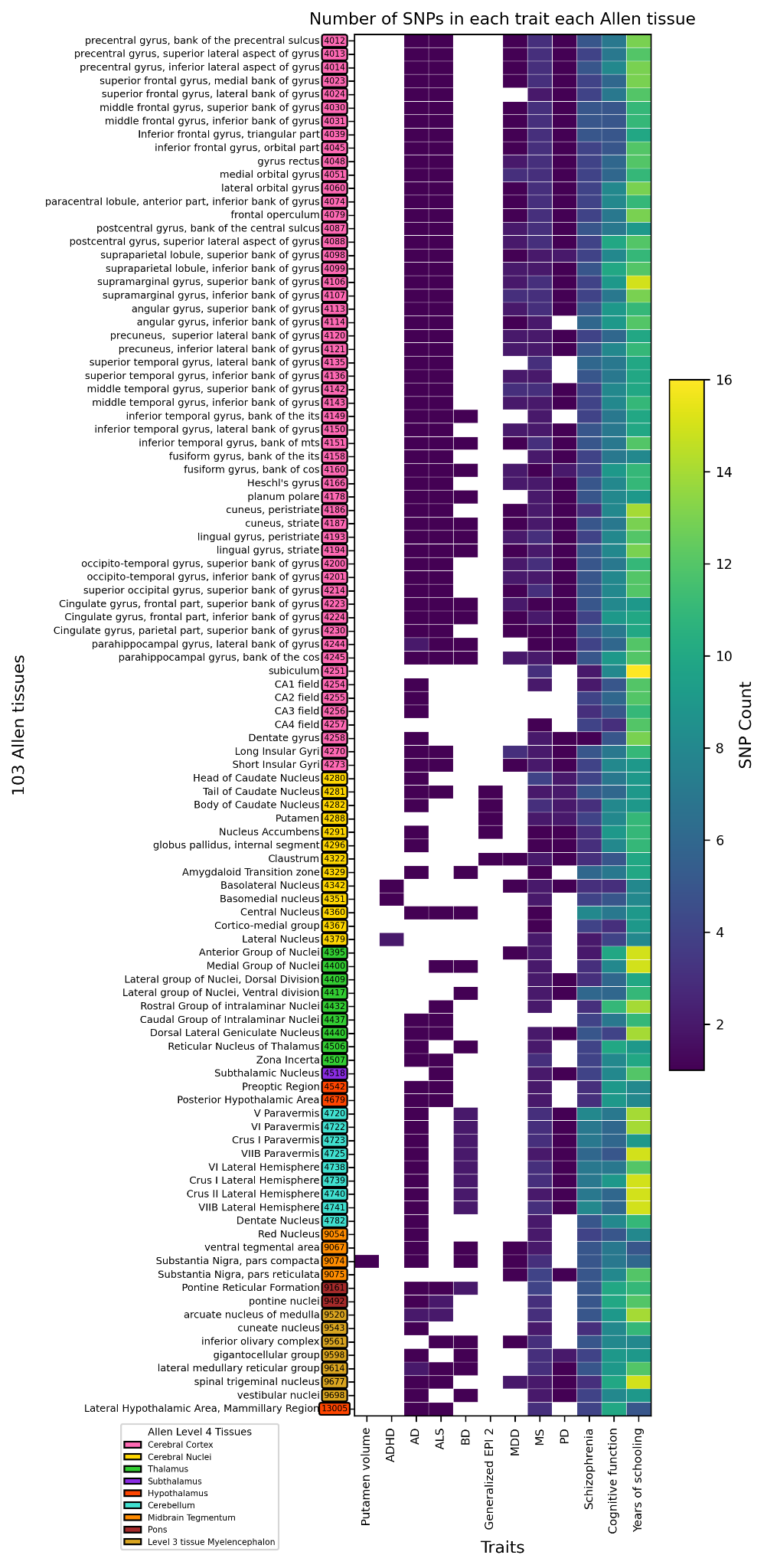

Supplement: Supplement 1 [file media-1.zip › Supplementary Figure 4.docx]
